# Cell therapy for stroke: use of local astrocytes

**DOI:** 10.3389/fncel.2012.00049

**Published:** 2012-10-31

**Authors:** Melek Chouchane, Marcos R. Costa

**Affiliations:** Brain Institute, Federal University of Rio Grande do NorteNatal, Brazil

**Keywords:** stroke, neuronal replacement, astrocyte reprogramming

## Abstract

Stroke refers to a variety of conditions caused by the occlusion or hemorrhage of blood vessels supplying the brain, which is one of the main causes of death and the leading cause of disability worldwide. In the last years, cell-based therapies have been proposed as a new approach to ameliorate post-stroke deficits. However, the most appropriate type of cell to be used in such therapies, as well as their sources, remains a matter of intense research. A good candidate cell should, in principle, display high plasticity to generate diverse types of neurons and, at the same time, low risk to cause undesired outcomes, such as malignant transformation. Recently, a new approach grounded on the reprogramming of endogenous astrocytes toward neuronal fates emerged as an alternative to restore neurological functions in several central nervous system diseases. In this perspective, we review data about the potential of astrocytes to become functional neurons following expression of neurogenic genes and discuss the potential benefits and risks of reprogramming astrocytes in the glial scar to replace neurons lost after stroke.

## Background

Ischemic insults result in a severe loss of neural cells in the core of the lesion and variable effects in the surrounding area, commonly described as ischemic penumbra. While cell death occurs during the first hours after interruption of blood supply within the core of the ischemic lesion, tissue damage in the surrounding regions is a delayed process, involving several physiopathological events, such as inflammation and immune response (for a review see Doyle et al., 2008). Treatments aiming to increase neuronal survival after stroke are usually limited to the area of penumbra and their success in promoting functional recovery is largely dependent on the extension of the core ischemic area (Goldstein, 2007). Currently, the only treatment available to reduce the size of the ischemic area is the use of recombinant tissue plasminogen activator (t-PA) (1995), which is approved to be administered within 3 h after the onset of ischemia (Goldstein, 2007). This narrow time window as well as a number of contraindications for t-PA therapy makes such treatment accessible to an extremely low number of stroke victims (Katzan et al., 2000), boosting the necessity to develop new strategies to treat stroke patients.

In the last years, cell-based therapies have been proposed as a new approach to ameliorate post stroke deficits. Different from t-PA, cell-based therapies could, in principle, be administered at any time following the ischemic event and contribute to replace neurons lost after ischemia and presumably restore neurological functions (Lindvall and Kokaia, 2011). Chiefly, two main types of intervention have been proposed: (1) transplantation of exogenous cells (Benchoua and Onteniente, 2011; Lindvall and Kokaia, 2011); and (2) mobilization of endogenous stem or progenitor cells (Leker et al., 2009; Lindvall and Kokaia, 2011). Both strategies have been shown to promote some degree of improvement in animal models of stroke (Lindvall and Kokaia, 2004, 2006). Yet, important limitations regarding the number of neurons replaced, specification of these neurons into the appropriate neurochemical subtypes, integration to the preexisting circuitry and potential side effects hamper the translation into clinical practice of such therapies.

Amongst the potential side effects, the most feared is the generation of tumors. In fact, it has been shown that both stem cell transplantation and stimulation of endogenous neural stem cell proliferation can lead to tumor formation in rodents and humans (Doetsch et al., 2002; Erdo et al., 2003; Amariglio et al., 2009). Therefore, the development of new strategies to replace neurons following stroke circumventing the limitations and risks discussed above is imperative to move cell-based therapies into clinic. In this scenario, we put in perspective the potential of a new approach grounded on the reprogramming of local astrocytes into neurons.

## Astroglial cells in the adult brain possess neurogenic potential

Contrary to the previous notion that neurons were not generated in the mammalian brain after birth, in the last two decades two neurogenic regions in the adult mammalian brain have been uncovered: the subependymal zone (SEZ), located along the lateral walls of the lateral ventricles, which holds a population of astroglial neural stem cells (ANSC) that constantly supply the olfactory bulb with interneurons (Kriegstein and Alvarez-Buylla, 2009); and the subgranular zone (SGZ) of the hippocampus, which also contains a population of ANSC capable of generating neurons to the dentate gyrus throughout life (Gage, 2000). Besides the significance of these findings to our understanding of brain physiology (Lledo et al., 2006), they also opened a new and promising avenue to brain repair after damage (Lindvall and Kokaia, 2006).

In fact, it has been shown that global and focal ischemic injuries in rodents lead to a significant increase in the number of neurons generate from ANSC both in the SGZ and the SEZ (Liu et al., 1998; Kee et al., 2001; Arvidsson et al., 2002; Parent et al., 2002). Some of these newly generate neurons change their route of migration and roam to lesioned areas after focal ischemia, where they acquire some characteristics of local neurons (Arvidsson et al., 2002; Parent et al., 2002). However, survival of these neurons in the lesioned areas is extremely poor, suggesting that some survival signal might be missing in re-routed neurons, leading to their premature death. As mentioned before, treatments with growth factors, used to increase the generation and survival of newborn neurons from endogenous neural stem cells (Leker et al., 2009), have also been related with glioma formation (Doetsch et al., 2002), making such approaches unsafe. Moreover, it remains to be shown whether such approach could be of any use in humans, given the great distance that newly generated neurons would need to transverse to reach the lesioned cortical tissue as compared to the rodent brain. Last but not least, the functionality of therapies aiming to recruit neurons from endogenous neurogenic niches relies on the occurrence of neurogenesis in the adult human brain, what has not been observed under physiological conditions (Sanai et al., 2004, 2011).

More recently, astrocytes of the cortical parenchyma, another population of astroglial cells, were suggested as an alternative source for neuronal replacement in neurological diseases (Robel et al., 2011). Compared to ANSC residing in neurogenic compartments, cortical astrocytes would have four main advantages: (1) they are located within the lesioned site, eliminating the need of relocation; (2) their amount is significantly increased after stroke (Buffo et al., 2008), generating a large amount of exploitable cells; (3) they can be efficiently reprogrammed into neurons using simple molecular manipulations (Berninger et al., 2007; Heinrich et al., 2010; Blum et al., 2011); and (4) they are involved in the formation of the glial scar, which contributes to generate an anti-neurogenic environment (Pekny and Nilsson, 2005). Therefore, astrocyte reprogramming could provide at once a source of new neurons in large numbers to replace the circuitry lost after stroke and reduce some negative effects of the glial scar (see below).

## Efficient reprogramming of astrocytes into glutamatergic and GABAergic neurons

Astrocytes isolated from rodent postnatal brain are highly susceptible to neuronal reprogramming following forced expression of a single neurogenic fate determinant, such as Neurogenin 2 (NEUROG2), Distal-less homeobox 2 (DLX2), or Achaete-scute homolog 1 (ASCLl1, also known as MASH1) (Berninger et al., 2007; Heinrich et al., 2010; Blum et al., 2011). Interestingly, expression of NEUROG2 induces a glutamatergic phenotype whereas the expression of MASH1 and DLX2 induces a GABAergic phenotype, resembling the roles of those transcription factors (TFs) in the developing forebrain (Guillemot, 2005). Astrocytes can not only be reprogrammed into neurons of specific subtypes but also acquire electrical properties compatible with a mature neuronal phenotype, such as intrinsic excitability and the ability to generate action potentials and synaptic contacts (Berninger et al., 2007; Heinrich et al., 2010).

**Figure 1:**
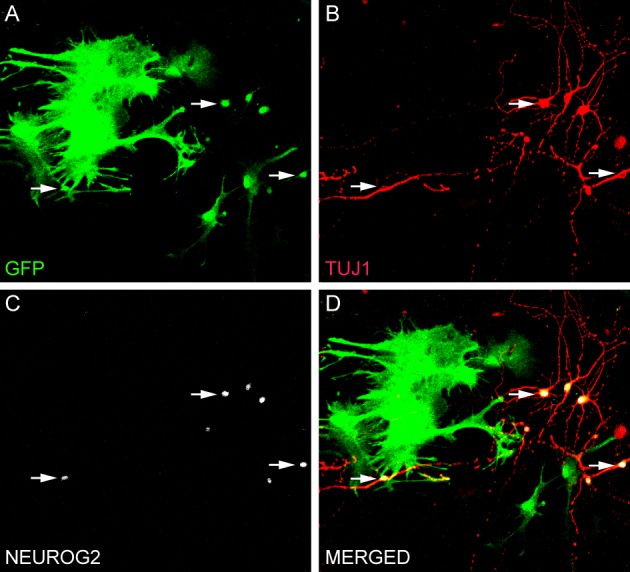
**Direct conversion of astrocytes into neurons *in vitro*. (A–D)** Culture of astrocytes isolated from the postnatal cerebral cortex of transgenic mice expressing green fluorescent protein (GFP) under the control of the astrocyte-specific promoter hGFAP (Nolte et al., 2001). Astrocytes were transduced with NEUROG2 2 h after plating and processed for immunocytochemistry after 7 days using antibodies against GFP (green, **A** and **D**), NEUROG2 (white, **C** and **D**) and the neuronal marker TUJ1 (red, **B** and **D**). Observe that astrocytes transduced with NEUROG2 (three of these cells are highlighted with arrows) adopted a neuronal phenotype, but still express residual levels of GFP, indicating their astrocytic origin.

Reprogramming of postnatal astrocytes using neurogenic TFs is a highly efficient process. Approximately, 70% of NEUROG2-transduced astrocytes differentiated into βIII tubulin-positive neurons after 7–10 days (Figure 1; Berninger et al., 2007; Heinrich et al., 2010). By 2–3 weeks post-transduction, reprogrammed neurons acquire MAP2 immunoreactivity, indicative for dendritic maturation, and express the vesicular glutamate transporter 1 (VGLUT1), present in synaptic vesicles within presynaptic terminals of glutamatergic neurons (Heinrich et al., 2010). Thus, astrocytes reprogrammed by forced expression of a single TF (NEUROG2) adopt a full neuronal glutamatergic phenotype forming presynaptic specializations. Indeed, electrophysiological recordings of neurons reprogrammed from astrocytes with NEUROG2 demonstrated both autaptic and synaptic currents that were blocked by CNQX (an AMPA/kainate glutamate receptor antagonist), further confirming the glutamatergic nature of the reprogrammed neurons. Amongst all NEUROG2-transduced astrocyte-derived neurons recorded, ~60% exhibited either glutamatergic autaptic connections onto themselves or glutamatergic synapses onto nearby neurons (Heinrich et al., 2010). Calcium-imaging experiments demonstrated that cultures of astrocytes reprogrammed with NEUROG2 are even capable of generating networks of spontaneously active neurons (Heinrich et al., 2010; Blum et al., 2011).

The efficiency of reprogramming astrocytes into GABAergic neurons using MASH1 or DLX2 (~35%) is lower than into glutamatergic neurons using NEUROG2 (Berninger et al., 2007; Heinrich et al., 2010). Nevertheless, neurons reprogrammed from astrocytes using DLX2 express GAD67 immunoreactivity, display autaptic responses with slow decay time kinetics which are abolished by the GABAA receptor antagonist and show spontaneous synaptic currents exhibiting a slow decay time, characteristic of GABAergic current (Heinrich et al., 2010), indicating that astrocytes are converted to functional GABAergic neurons. Co-expression of MASH1 and DLX2 in postnatal astrocytes increased the rate of neuronal conversion up to 90% (Heinrich et al., 2010), showing that the efficiency to reprogram astrocytes into GABAergic neurons can be drastically improved by combining two TFs. Taken together, these data clearly indicate that astrocytes can be efficiently converted to functional glutamatergic or GABAergic neurons through simple molecular manipulations.

## Reprogramming of postnatal astrocytes into dopaminergic neurons

Postnatal cortical astrocytes can also be reprogrammed into dopaminergic neurons, although this requires a more complex set of TFs (Addis et al., 2011). Astrocytes-transduced with a polycistronic lentiviral vector encoding for MASH1, LIM homeobox TF 1 (LMX1), and nuclear receptor related 1 protein (NURR1) differentiate into neurons expressing biochemical and electrophysiological characteristics analogous with midbrain dopaminergic neurons. However, the efficiency of astrocyte conversion to dopaminergic neurons (~18%) is much lower than that described previously for glutamatergic and GABAergic neurons (Heinrich et al., 2010; Addis et al., 2011). Yet, the finding that astrocytes isolated from a region that normally do not generate dopaminergic neurons can be reprogrammed into these types of neurons using few TFs reveals the great plasticity of astrocytes and supports the notion that these cells are good candidates to replace distinct types of neurons in damaged brain areas.

## Astrocytes in the adult brain resume proliferation and acquire neurogenic potential after lesion

Astrocytes account for up to one-fifth of the dividing cells in the first 7 days following traumatic or ischemic brain injury (Buffo et al., 2005) and at least part of these cells are mature astrocytes that resume proliferation after lesion (Buffo et al., 2008). These reactive, proliferating astrocytes acquire some neural stem cell-like properties after injury (Buffo et al., 2008; Robel et al., 2011) and are suitable to reprogramming into functional neurons (Heinrich et al., 2010).

Although astrocyte activation may play beneficial roles at early time-points after stroke, there is convincing evidence that astrocytes in the glial scar are detrimental for regeneration of the adult brain (Pekny and Nilsson, 2005; Robel et al., 2011). For instance, attenuation of reactive gliosis through genetic deletion of intermediate filaments (IFs) glial fibrillary acidic protein (GFAP) and vimentin in animals subjected to traumatic brain injury improved regeneration, with a positive effect on complete synaptic restoration (Wilhelmsson et al., 2004). In these same models, neuronal differentiation and dendritic growth of transplanted cells were enhanced after transplantation, indicating that reactive gliosis adversely affects integration of neuronal cells (Widestrand et al., 2007). In an opposite direction, increasing reactive gliosis worsens brain injuries, as demonstrated by the finding that overexpression of S100b, an astrocyte-derived protein, enlarged infarct size and impaired neurological outcome after ischemia (Mori et al., 2008). Therefore, reprogramming of astrocytes in the glial scar could *per se* improve neurological functions after stroke.

In an ideal scenario, we should be able to find a balance between diminishing the number of detrimental astrocytes in the glial scar through reprogramming of these cells into neurons and, at the same time, conserve non-reprogrammed astrocytes that could contribute to create an appropriate environment for the development and functioning of new synaptic contacts between reprogrammed neurons and the pre-existing circuitry (Wang and Bordey, 2008). To this point, it is unclear whether reactive astrocytes acquiring stem cell-like properties after injury represent a sobpopulation of astrocytes and what would be the role of such cells in the glial scar. Future studies should help to clarify this point and indicate methods to target specific subpopulations of astrocytes to reprogramming.

## Reprogramming of human astrocytes into neurons

An important question toward translation of astrocyte reprogramming into clinic would be whether human astrocytes possess the same potential to be reprogrammed into neurons. A partial answer to this question has been recently published in a paper from Corti et al. (2012). By cultivating astrocytes from the human cerebral cortex and inducing the expression of TFs involved in pluripotency (Takahashi and Yamanaka, 2006; Wernig et al., 2007), the authors could show that astrocytes expressing OCT4, SOX2, or NANOG generated colonies of neural stem cells (Corti et al., 2012). These colonies could be expanded and differentiated into the three major neural cell types—neurons, astrocytes, and oligodendrocytes (Corti et al., 2012). Neurons expressed typical neuronal proteins, such as MAP2, synapsin and GABA, suggesting that human astrocytes could be reprogrammed into neurons acquiring part of the machinery to establish synaptic contacts. Expression of MASH1 in NSCs derived from human astrocytes significantly increased the frequency of neuronal differentiation (Corti et al., 2012), further supporting the key role of neurogenic determinants to convert astrocytes into neurons.

Strikingly, human astrocytes transduced with NANOG and transplanted in the lateral ventricles of immunosuppressed mice survived and integrated into the host brains 2 months after delivery. Some transplanted cells expressed MAP2 and displayed complex and long neuritic extensions, compatible with neuronal differentiation (Corti et al., 2012). Thus, human astrocytes can be efficiently reprogrammed into neurons both *in vitro* and *in vivo*. Noteworthy, neuronal conversion of human astrocytes occurred without regression to a pluripotent state, what could contribute to avoid some complications linked to that state, including the risk of malignancy.

## Reprogramming of astrocytes into subtype-specific neurons

**Figure 2:**
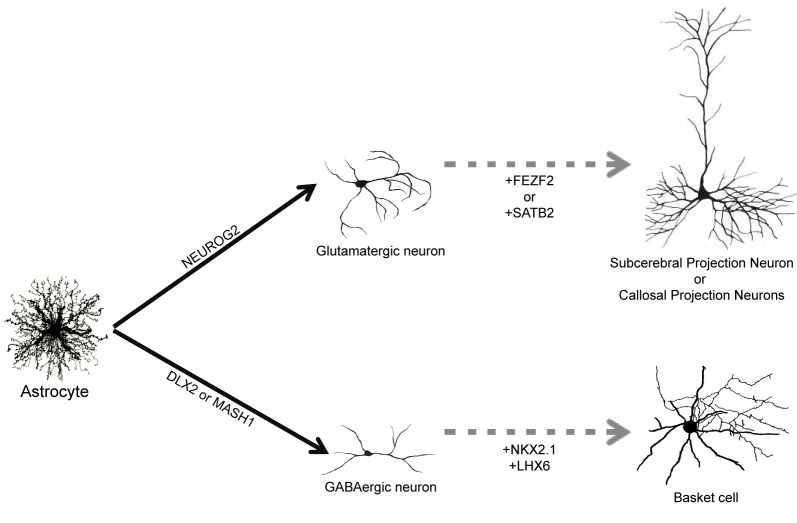
**Direct reprogramming of astrocytes into subtype specific neurons.** Astrocytes can be converted into glutamatergic neurons by forced expression of NEUROG2 and into GABAergic neurons following expression of DLX2 and MASH1 (filled arrows). Up to now, it is unknown which subtype of glutamatergic and GABAergic will be generated *in vivo*. We suggest that co-expression of additional TFs, such as FEZF2, SATB2 or NKX2.1/LHX6, could contribute to generate more specific subtypes of neurons such as subcerebral projection neurons, callosal projection neurons or basket cells, respectively (dashed arrows).

The adult human brain harbors a large variety of neuronal cell types, each exhibiting specific structural, molecular, and functional features (DeFelipe, 1993; Douglas and Martin, 2004; Markram et al., 2004; Klausberger and Somogyi, 2008). Therefore, one important step toward the clinical translation of astrocyte reprogramming as a therapy for stroke would be to direct the specification of defined neuronal subtypes. It remains to be evaluated, for instance, whether astrocytes reprogrammed by forced expression of NEUROG2 will generate principal and local glutamatergic neurons of different cortical layers. Similarly, it is unclear whether GABAergic neurons generated from astrocytes reprogrammed with MASH1, DLX2, or combination of these two TFs will adopt distinct morphological and electrophysiological properties, contributing to generate distinct subtypes of GABAergic interneurons, such as basket and chandelier cells (Markram et al., 2004) (Figure 2). These questions can only be answered by experiments assessing the differentiation of neurons reprogrammed from astrocytes *in vivo*, either by expressing neurogenic TFs in astrocytes directly in the brain or transplanting astrocytes previously transduced with neurogenic TFs *in vitro* into the healthy or injured brain. Such experiments will allow the evaluation of neuronal morphology, connectivity and synaptic formation adopted by reprogrammed astrocytes exposed to the brain environment.

Nevertheless, data from studies unraveling the molecular machinery responsible for the generation of neuronal diversity during development may help to suggest strategies to reprogram astrocytes into specific subtypes of neurons. In the last decade, several works have contributed to identify the genetic machinery involved in the specification of distinct populations of cortical glutamatergic neurons (Arlotta et al., 2005; Molyneaux et al., 2007; Leone et al., 2008). For example, family zinc finger 2 (FEZF2) is necessary for the specification of subcerebral projection neurons (Chen et al., 2005a,b; Molyneaux et al., 2005), whereas SATB homeobox 2 (SATB2) is required for proper specification of callosal projection neurons (Alcamo et al., 2008). It is tempting to speculate that co-expression of NEUROG2 and FEZF2 or SATB2 in astrocytes would drive reprogrammed neurons into subcerebral and callosal projection neurons, respectively (Figure 2). In accordance with this possibility, expression of FEZF2 in striatal progenitors during development is sufficient to generate cortifugal neurons (Rouaux and Arlotta, 2011).

Similarly, subtypes of cortical GABAergic interneurons originate from separate progenitor domains characterized by expression of distinct sets of TFs (Wonders and Anderson, 2006; Hernandez-Miranda et al., 2010). For instance, parvalbumine-expressing basket cells originate from progenitors in the medial ganglionic eminence that express the TFs NK2 homeobox 1 (NKX2.1) and LIM homeobox 6 (LHX6), whereas calretinin-expressing interneurons originate from the caudal ganglionic eminence areas that do not express NKX2.1 (Wonders and Anderson, 2006; Hernandez-Miranda et al., 2010). Therefore, it is also feasible that distinct subsets of cortical GABAergic neurons could be generated from astrocytes through the expression of specific combinations of TFs (Figure 2).

## Targeting astrocytes for reprogramming *in vivo*

Finally, for future clinical approaches, two additional points remain to be elucidated. The first is how to target specifically astrocytes within the glial scar, avoiding reprogramming of other astrocytic populations. The second point is how to deliver reprogramming factors to astrocytes *in vivo*. As we discussed above, techniques to convert astrocytes into neurons rely on genetic manipulations of the cells, which may cause unexpected genetic modifications (Yamanaka, 2007). One possible way to circumvent the need to introduce exogenous genes in astrocytes could be the use of recombinant proteins. This has been done to convert fibroblasts into induced pluripotent stem cells (Zhou et al., 2009) and it is likely to work in astrocytes. Another possibility would be the use of molecules capable of modifying extracellular signals involved in cell specification. Kondo and Raff (2000), for instance, have shown that oligodendrocytes precursor cells isolated from the optic nerve and cultured sequentially in platelet derived growth factor (PDGF), fetal calf serum (FCS), and basic fibroblast growth factor (bFGF), could revert to a multipotent neural stem cell state and differentiate into neurons (Kondo and Raff, 2000). It is tempting to speculate that pharmacological manipulations of the extracellular milieu or use of recombinant proteins could substitute DNA elements needed for reprogramming of astrocytes in the glial scar and, therefore, represent a safe strategy to replace neurons in human patients after stroke.

## Conclusions

Reprogramming of astrocytes in the glial scar into neurons is a promising approach toward regeneration of nervous tissue after stroke. In contrast to stem cell transplantation and recruitment of neural stem cells from neurogenic regions in the adult brain, parenchymal astrocytes possess the advantage of being present in large amounts around the lesion. Moreover, conversion of reactive astrocytes into neurons would not only contribute to replace neuronal populations lost but also help to create an environment more suitable for neuronal growth and synaptic integration.

Future studies should clarify the potential of reprogrammed astrocytes to generate different subtypes of neurons *in vivo*, as well as identify the subpopulations of astrocytes more suitable to reprogramming after stroke. Simultaneously, TFs networks capable of reprogramming astrocytes into specific subtypes of neurons should be identified, contributing to design more sophisticated approaches to selectively replace neuronal populations lost after stroke.

### Conflict of interest statement

The authors declare that the research was conducted in the absence of any commercial or financial relationships that could be construed as a potential conflict of interest.
